# Digital-Supported Delivery of Behavioural Therapy for Patients with Tic Disorders: A Narrative Review

**DOI:** 10.3390/brainsci16050453

**Published:** 2026-04-24

**Authors:** Kamila Saramak, Anna Dunalska, Katarzyna Śmiłowska, Wiktor Śliwiński, Ali Abusrair, Sanja Gluščević, Simon Schmitt, Kirsten R. Müller-Vahl, Natalia Szejko

**Affiliations:** 1 Department of Neurology, Medical University of Innsbruck, 6020 Innsbruck, Austria; kamila.saramak@tirol-kliniken.at; 2 Department of Psychiatry, Faculty of Health Sciences, Medical University of Warsaw, 02-091 Warsaw, Poland; 3 Department of Neurology, 5th Regional Hospital in Sosnowiec, 41-200 Sosnowiec, Poland; 4 The Independent Public Healthcare Institution of the Ministry of the Interior and Administration, 90-521 Lodz, Poland; 5 Neurology Division, Department of Internal Medicine, Qatif Health Network, Qatif 31911, Saudi Arabia; abusrair.md@gmail.com; 6 Clinical Centre of Montenegro, Neurology Clinic, 81000 Podgorica, Montenegro; 7 Department of Psychiatry, Psychotherapy and Psychosomatics, RWTH Aachen University, 52074 Aachen, Germany; sischmitt@ukaachen.de; 8 Clinic of Psychiatry, Social Psychiatry and Psychotherapy, Hannover Medical School, 30625 Hannover, Germany; mueller-vahl.kirsten@mh-hannover.de; 9 Department of Health Sociology, Education and Medical Communication Institute of Mother and Child, 01-211 Warsaw, Poland

**Keywords:** Tourette syndrome, tic disorders, behavioral therapy, digital health, tic detection, comprehensive behavioral intervention for tics, habit reversal training, HRT, CBIT, iCBIT

## Abstract

**Highlights:**

**What are the main findings?**

- Digital and telehealth-delivered behavioural therapies (CBIT, HRT, and ERP) for tic disorders are feasible, safe, and show efficacy comparable to face-to-face treatment across age groups.
- Internet- and app-based interventions reduce tic severity, improve accessibility, and require less therapist time while maintaining high patient satisfaction and adherence.

**What are the implications of the main findings?**

- Digitally delivered behavioural therapy has the potential to significantly reduce the treatment gap in tic disorders by overcoming geographical, logistical, and workforce limitations.
- Future implementation should focus on standardized protocols, long-term outcomes, and personalized approaches to optimize effectiveness across diverse patient populations.

**Abstract:**

Background: Behavioural therapy (BT), including Comprehensive Behavioural Intervention for Tics (CBIT), is an evidence-based first-line treatment for patients with tic disorders. However, access remains limited due to a shortage of trained providers, geographical barriers, costs, and high treatment burden for patients and families. Rapid advances in digital health technologies including telemedicine, web-based treatment platforms, and mobile applications offer new opportunities to expand access to BT for individuals with tic disorders across the lifespan. Methods: For the purpose of this narrative review, we conducted a literature search in PubMed, Europe PMC, and the Cochrane Library to identify relevant studies investigating the effectiveness of digital health treatment approaches in tic disorders. Results: A total of 16 original studies were included. Although the available evidence remains limited and heterogeneous, existing studies suggest that emerging technologies for delivering behavioural interventions for tic disorders, including telehealth-based CBIT, digital therapy platforms, and app-supported habit reversal training (HRT), are feasible, cost-effective, user-friendly, flexible, and safe. These approaches also appear effective for symptom monitoring and personalized treatment support in both pediatric and adult populations. Conclusions: Recent technological advances have the potential to reduce the treatment gap in tic disorders, provided that these approaches are implemented within rigorous, evidence-based, and ethically grounded frameworks.

## 1. Introduction

Tics are defined as brief, purposeless, and unintentional movements or noises, which are often preceded by premonitory urges and can be temporarily suppressed, albeit at the cost of increasing internal discomfort [1]. Primary tic disorder shows a heterogeneous spectrum, ranging from provisional and chronic motor or vocal tic disorders to Tourette syndrome (TS), which represents the most severe and complex phenotype [2]. TS is characterized by the presence of multiple motor tics and at least one vocal tic, with onset in childhood and a fluctuating course over time [3]. Chronic tic disorders are frequently accompanied with psychiatric comorbidities, most commonly attention-deficit/hyperactivity disorder (ADHD), obsessive–compulsive disorder (OCD), depression, anxiety, autism spectrum disorder (ASD), and self-injurious behaviour (SIB) [4,5,6,7]. These associated conditions substantially contribute to functional impairment and reduced quality of life in affected individuals [8]. Regarding available treatments, both the European Society for the Study of Tourette Syndrome (ESSTS) [9] and the American Academy of Neurology (AAN) [10] recommend psychological interventions and/or pharmacotherapy as first-line management of tics. Based on clinical consensus, psychoeducation is acknowledged as an initial therapeutic step regardless of tic severity according to the ESSTS guidelines [9]. If psychoeducation alone turns out to be insufficient, behavioural therapy (BT) including Comprehensive Behavioural Intervention for Tics (CBIT), habit reversal training (HRT) as well as exposure and response prevention (ERP) should be considered [11]. Pharmacological therapy (PT) is primarily based on dopamine receptor blockade [12]. For those patients who do not benefit from standard treatments or experience serious adverse effects (AEs), experimental therapies such as cannabis-based medicine can be considered [13,14,15].

Psychoeducation involves the structured provision of clear, accessible, and current information about a condition, including its symptoms, underlying mechanisms, prognosis, treatment options, management strategies, and impact on daily life [16]. When used in the context of TS, psychoeducation typically addresses the nature and classification of tics (e.g., motor vs. vocal, simple vs. complex, and phenomena such as coprolalia), the typical developmental course of tic disorders, and the role of premonitory urges—uncomfortable internal sensations that precede tics and are temporarily relieved by performing the tic. It may also explain related sensory experiences such as “just-right” feelings and other internal factors that influence tic expression and maintenance [17,18]. Although psychoeducation is consistently recommended in clinical guidelines as an essential initial component of treatment for patients with tic disorders, there is limited evidence defining which specific elements should be included to achieve optimal clinical benefit [9]. Moreover, psychoeducation has been shown to be less effective than BT and pharmacological treatment in several randomized controlled trials (RCTs) [11,19,20]. Among behavioural interventions, HRT and its expanded form, CBIT, are the most extensively studied and evidence-based approaches [11,21,22,23]. HRT includes awareness training to increase recognition of tics and premonitory urges, followed by competing response training, during which incompatible responses are practiced to prevent tic expression. In this approach, tics are typically addressed individually, with patients first identifying and monitoring their different tics and selecting the most bothersome one to target initially. During awareness training, individuals learn to detect the tic and the early internal signals that precede it. Once sufficient awareness is developed, a competing response, usually involving the same muscle groups and capable of being maintained for a short period, is practiced to inhibit the tic and later rehearsed outside therapy sessions [24,25]. CBIT is a multicomponent behavioural treatment that builds upon the core techniques of HRT while addressing environmental and psychological factors that may exacerbate tics. In addition to awareness and competing response training, CBIT includes relaxation techniques to reduce stress and physiological arousal, which are known to influence tic severity. The intervention also incorporates contingency management strategies that guide parents, teachers, or caregivers in responding to tics in ways that minimize reinforcement of tic behaviours [26]. Another BT approach is ERP, which, like HRT, is grounded in learning theory and involves systematic suppression of tics for progressively increasing durations while exposing individuals to premonitory urges and tic-provoking situations. In contrast to HRT, ERP addresses all tics simultaneously without employing a tic hierarchy, with the goal of increasing urge tolerance and thereby reducing tic expression [27].

Despite their proven efficacy, access to these interventions remains limited due to a shortage of trained therapists, geographical barriers, increasing costs, and the substantial treatment burden for families. These challenges, together with the public crisis during the COVID-19 pandemic, have driven the development of digital and remote delivery formats, including telemedicine and internet-based interventions to expand their reach to a larger proportion of the population. The aim of this article is to summarize the current evidence on remotely delivered BT for tic disorders across the lifespan and to provide an update of previously published reviews [28,29,30]. In addition, given the limited number of RCTs, we aimed to extend beyond existing systematic reviews by including open-label, uncontrolled studies and by outlining future directions through the discussion of preliminary findings and ongoing studies. Specifically, we address the following questions: (1) how effective are digital and telehealth-based interventions (CBIT, HRT, and ERP) in reducing tic severity?; (2) how do these approaches compare with face-to-face treatment in terms of feasibility, acceptability, and adherence?; and (3) what are the key limitations and future directions for their clinical implementation? The findings are structured into the following three sections: videoconference-based therapy, internet- and app-based interventions, and a discussion of clinical implications and future perspectives.

## 2. Materials and Methods

For the purpose of this narrative review, we followed SANRA recommendations for narrative reviews [31]. A literature search was conducted independently by two authors (KS, AD) to identify studies investigating digital or technology-supported behavioural interventions for tic disorders. Discrepancies were resolved by a senior reviewer (NS). The search was performed in PubMed, Europe PMC, and the Cochrane Library. The following search terms were used: “Tourette syndrome” [MeSH Terms] OR “Gilles de la Tourette syndrome” [MeSH Terms] OR “tic disorders” [MeSH Terms] OR “tics” [MeSH Terms] AND “behavioural therapy” [Title/Abstract].

Eligible studies included original studies and case reports conducted in humans and published in English that investigated digital, telemedicine-based, or app-supported behavioural interventions for tic disorders in pediatric and/or adult populations. Outcomes of interest included tic severity, feasibility, treatment adherence, and patient satisfaction. RCTs, randomized crossover trials, and uncontrolled studies were included. In addition, reference lists of all included articles were manually screened to identify further relevant studies. No formal risk-of-bias or methodological quality assessment was conducted beyond the standard peer-review process, in line with the narrative design of this review. Nevertheless, the limitations of the included studies are addressed within Section 4. No time restrictions were applied, and the final search was conducted in March 2026.

**Figure 1:**
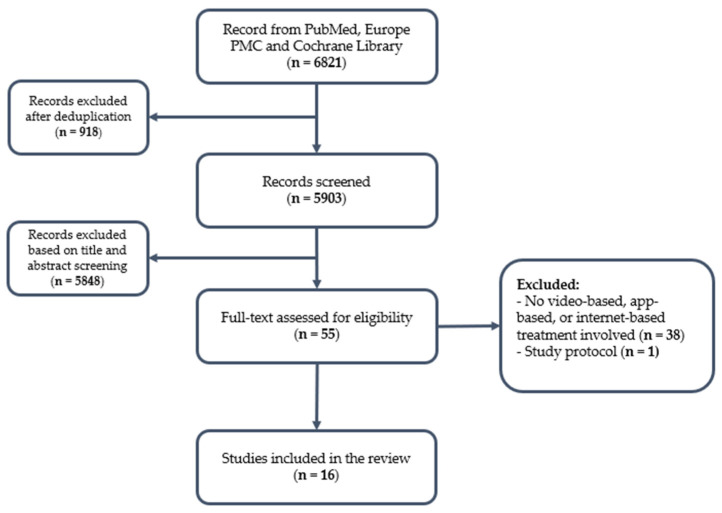
Flow diagram of the studies included in the review.

Due to heterogeneity in study design and outcome reporting, results were summarized descriptively. The selection process is summarized in Figure 1.

## 3. Results

In total, we identified 16 original studies (11 RCTs, two randomized crossover trials, one single-arm open-label trial, one observational comparative study and one case series) focusing on the remote delivery of BT for patients with tic disorders. To provide a structured overview of the available evidence, the results are organized according to the mode of remote delivery, distinguishing between videoconference-based interventions and internet-based approaches.

### 3.1. Videoconference Delivery of Behavioural Therapy

Videoconference-delivered BT closely mirrors standard face-to-face (F2F) treatment, with the primary distinction being that real-time communication between the patient and therapist is facilitated through videoconferencing software. Himle et al. [32] conducted the first RCT directly comparing videoconference-delivered CBIT (tele-health CBIT) with F2F BT in 20 children. Ten children received telehealth CBIT and ten received F2F treatment. The intervention consisted of eight sessions held almost weekly, delivered over a 10-week period. Both treatment formats resulted in significant reductions in tic severity as measured by the Total Tic Severity Score of the Yale Global Tic Severity Scale (YGTSS-TSS), with no between-group differences at the 4-month follow-up; mean YGTSS-TSS scores decreased by 7.8 points (33%) in the telehealth group and by 6.5 points (27%) in the F2F group. Regarding secondary outcomes, based on the Clinical Global Impression–Improvement scale (CGI-I), 80% of children in the telehealth condition and 75% in the F2F condition were rated as treatment responders. Treatment acceptability and therapeutic alliance were rated highly in both conditions, and there were no statistically significant differences between the groups.

Subsequently, Ricketts et al. [33,34] compared CBIT with a waitlist control (WL) condition in a sample of 20 children with tic disorder. Of the 20 participants enrolled, 12 were randomized to CBIT delivered via voice over internet protocol (CBIT-VoIP), and eight were assigned to the WL condition. Both the child and the parent were required to attend sessions; however, this requirement was waived for mature older adolescents. The treatment was delivered over a 10-week period. At post-treatment, significantly greater reductions in tic severity were observed in the CBIT-VoIP group compared with the WL condition, as measured by the clinician-rated YGTSS-TTS and parent-reported Parent Tic Questionnaire (PTQ) scores. Participants receiving CBIT-VoIP showed a mean reduction of 7.25 points (28.2%) in YGTSS-TTS scores. One-third of participants in the CBIT-VoIP group was classified as treatment responders. Although minor technical challenges were reported, including occasional audio–video issues and difficulties reviewing therapeutic homework assignments, high levels of therapeutic alliance, treatment satisfaction, and overall satisfaction with videoconferencing were observed.

Singer et al. [35] evaluated a home-based, parent-administered HRT programme supported by instructional video materials and a parent manual. In this study, 33 children were assigned to the video-based intervention and 11 to therapist-led, F2F HRT. Eighteen participants completed the study, including eight in the video-HRT group and 10 in the F2F HRT group. Participants in the therapist-led condition attended eight sessions over a 10-week period, conducted weekly during the first six weeks and biweekly at weeks eight and 10. At the 10-week post-treatment assessment, both groups demonstrated statistically significant improvements, reflected by substantial reductions in YGTSS scores. Mean TSS improvements were 9 points (32.4%) in the video group and 7.5 points (26.6%) in the in-person group, with corresponding Global Severity Score improvements of 33.7% and 26.7%, respectively. Among the 18 individuals who completed the 10-week treatment, the improvement observed in the video group did not differ significantly from that of the F2F group.

In a subsequent RCT, Prato et al. [36] assigned 40 children and adolescents with TS to receive BT either F2F or remotely via therapist-delivered videoconferencing (VoIP). Twenty participants received VoIP-BT and 20 received F2F-BT. The intervention consisted of HRT or ERP delivered across eight weekly sessions. Both treatment groups demonstrated statistically significant reductions in tic severity, as measured by the YGTSS, two months after randomization. The mean total reduction in YGTSS-TSS was 11.4 points (44.7%) in the remote BT group and 12.05 points (46.8%) in the F2F-BT group. No statistically significant differences were observed between the groups in the change in tic severity from baseline to the two-month follow-up. In addition, both groups showed statistically significant improvements in obsessive–compulsive symptoms and anxiety levels, as measured by the Children’s Yale–Brown Obsessive Compulsive Scale (CY-BOCS) and the Multidimensional Anxiety Scale for Children (MASC), with no significant differences between groups. Notably, VoIP-BT was associated with a significantly greater reduction in depressive symptoms, as assessed by the Child Depression Inventory (CDI). The mean decrease in CDI scores at two months was 1.05 points (23.6%) in the remote BT group compared with 0.05 points (1.16%) in the F2F-BT group, suggesting potential additional benefits of remote delivery beyond tic reduction.

Capriotti et al. [37] evaluated the effectiveness of remotely delivered CBIT when integrated into routine care within specialized tic clinics in an open, uncontrolled study including 19 pediatric and 10 adult patients. Participants received eight manualized CBIT sessions delivered via videoconference during their usual care appointments. Outcomes were assessed at baseline, at post-treatment, and at 3- and 6-month follow-up by an independent rater. Statistically significant reductions in YGTSS-TTS of 6.84 points (25.95%) and 4.5 points (22%) were observed in the pediatric and adult populations, respectively. A positive global treatment response measured using the CGI-I was achieved by 68% of pediatric patients and 60% of adults at post-treatment. These findings support remote CBIT as an effective method for delivering evidence-based BT across age groups in clinical settings. Remote CBIT was also well-accepted: all participants who initiated treatment completed the full intervention and reported high satisfaction, and the vast majority of sessions occurred without technical difficulties.

In a subsequent open uncontrolled case series, Inoue et al. [38] examined the feasibility of remotely delivered, group-based CBIT in three children with TS. The intervention was conducted entirely online in a group format over a 10-week period and consisted of eight weekly sessions delivered via videoconferencing software, supplemented by slide-based instructional materials and a cloud-based learning platform. All participants demonstrated reductions in tic severity, with the YGTSS-TTS decreasing by an average of 7.0 points five days after completion of the intervention. Treatment adherence was complete, and no dropouts were reported, supporting the feasibility of remote group-based CBIT delivery.

On the other hand, Soerensen et al. [39,40] examined a less-researched BT approach, ERP, by comparing the efficacy of ERP delivered via web-based videoconferencing with traditional F2F sessions during treatment and at a one-year follow-up. A total of 116 children and adolescents were included, with 72 receiving in-person ERP and 44 treated via videoconference. There was a statistically significant decrease in the YGTSS-TTS over the course of treatment in both groups, and these improvements were maintained at the one-year follow-up. The mean total reduction in YGTSS-TTS was 11.7 points (46.4%) in the remote BT group and 10.1 points (46.8%) in the F2F-BT group. No significant differences in YGTSS-TTS were observed between the two delivery formats at baseline, at the end of treatment, or at the one-year follow-up, indicating that videoconference-based ERP achieves outcomes comparable to F2F therapy.

Recently, an RCT by Jöhnk et al. [41] compared therapist-guided, mobile app-assisted BT for children and adolescents with tics with videoconference-delivered BT. The study included 30 youths with TS or chronic tic disorder and their parents. Participants were randomized to receive eight sessions of either mobile app-assisted BT (*n* = 14) or videoconference-delivered BT (*n* = 16). The primary outcomes were feasibility, acceptability, adherence, and safety. As both interventions were considered feasible and acceptable by participating families, the primary outcomes were met. Moreover, both groups showed statistically significant reductions in tic severity at the end of treatment, as measured by the YGTSS-TTS, with mean reductions of 9.25 points in the app-assisted group and 7.43 points in the videoconference group. Participants in both groups experienced additional improvements in tic severity during the two-month follow-up period, with further reductions of 5.75 points in the app-assisted group and 5 points in the videoconference group.

All studies examining video-based and videoconference-based delivery of BT with further details are summarized in Table 1.

### 3.2. Internet Delivery of Behavioural Therapy

In internet-delivered BT, patients engage in a structured online self-help programme with limited therapist support provided via text messages or telephone. This treatment format was first implemented by the Swedish internet platform Barninternetprojektet (BIP), which has successfully delivered internet-based interventions for several pediatric mental health conditions, including tic disorders. The platform was also used by Andrén et al. [42] in 2019 to evaluate two therapist-guided, internet-delivered interventions based on habit reversal training (BIP TIC HRT) and exposure and response prevention (BIP TIC ERP) principles in a RCT involving 23 children and adolescents with tic disorders. Twelve patients were allocated to the ERP group and 11 to the HRT group. Both interventions led to statistically significant reductions in tic-related impairment and parent-rated tic severity. Nevertheless, only BIP TIC ERP resulted in a statistically significant improvement in clinician-rated tic severity, as measured by the YGTSS-TTS, at both the 3- and 12-month follow-ups. At 12 months, a reduction in YGTSS-TTS of 6.8 points (28.8%) was observed in this group. Importantly, both interventions required substantially less therapist time than traditional F2F BT, representing an additional practical advantage.

Subsequently, Andrén et al. [43] aimed to evaluate the efficacy of therapist-guided, internet-delivered ERP compared to psychoeducation in an RCT involving 221 children and adolescents with TS and chronic tic disorders. Participants were randomized to 10 weeks of therapist-supported internet-delivered ERP for tics (*n* = 111) or to therapist-supported internet-delivered education for tics (comparator group, *n* = 110). Both interventions resulted in statistically significant reductions in tic severity, as measured by the YGTSS-TTS, with no significant differences observed between the groups. At the 3-month follow-up, the mean reduction in YGTSS-TTS was 6.08 points in the ERP group and 5.29 points in the comparator group.

The same participant groups were evaluated at 12-month follow-up [44]. Based on YGTSS-TTSS scores, no statistically significant changes in tic severity were observed between the 3-month and 12-month follow-up in either group. A secondary analysis incorporating all time points from baseline to 12 months likewise showed no statistically significant difference between groups in tic severity over this period. Treatment response rates at 12 months were comparable (55% in ERP vs. 50% in psychoeducation). From a healthcare perspective, ERP yielded more quality-adjusted life years and lower costs than psychoeducation at 12 months.

To date, the largest RCT evaluating the efficacy of internet-delivered BT in the form of ERP was conducted by Hollis et al. [45]. This study investigated a therapist-supported, parent-assisted internet-delivered ERP programme for the treatment of tics in children and adolescents with TS or other chronic tic disorders. A total of 224 participants were randomly assigned in equal numbers to receive either 10 weeks of ERP or an internet-delivered psychoeducation intervention, with both treatments delivered via the secure online platform. The primary outcome was tic severity at 3 months post-randomization, assessed using the YGTSS-TTS. At this time point, a statistically significant between-group difference favouring ERP was observed, as mean total decrease in YGTSS-TTSS was 4.5 (16%) in the ERP group versus 1.6 (6%) in the psychoeducation group. The adjusted treatment effect on tic severity increased slightly between the 3- and 6-month follow-up assessments.

The long-term follow-up of the ORBIT trial by Hollis et al. [45] examined the durability of internet-delivered ERP compared with internet-delivered psychoeducation at 12 and 18 months after treatment initiation. Reductions in tic severity as measured by YGTSS-TTS were maintained over time in both treatment groups. Parent-reported tic severity, assessed using the PTQ, was significantly lower in the ERP group at the 12-month follow-up; however, this difference was no longer evident at 18 months. Overall, these findings indicate that internet-delivered ERP is associated with sustained clinical benefits for tic disorders for up to 18 months following treatment [46]. A subsequent economic evaluation demonstrated that delivering ERP online with minimal therapist involvement is an effective and cost-efficient treatment approach, with ERP associated with a cost difference of £304.94 compared with psychoeducation and a cost of £16,708 per quality-adjusted life year (QALY) gained at 18 months.

Rachamim et al. [47] investigated a digitally delivered, parent-guided self-help version of CBIT involving minimal therapist internet-delivered CBIT (iCBIT) and compared it with a WL. In this randomized crossover trial, 41 children and adolescents with tic disorders were assigned to either the iCBIT programme (*n* = 25) or the WL condition (*n* = 16). Participants receiving iCBIT demonstrated a substantial advantage over the WL in reducing tic severity, as assessed by the YGTSS-TTS, with 64% showing clinically statistically significant improvement at post-treatment. In addition to tic reduction, the intervention was associated with improvements in overall impairment and functioning, which were maintained at the 6-month follow-up. Notably, the program required markedly less therapist time than conventional F2F BT, highlighting its practicality and potential for broader implementation.

Building on their initial RCT, Rachamim et al. [48] conducted a secondary analysis to examine both short- and long-term outcomes of internet-delivered iCBIT in 38 youths with tic disorders, of whom 16 had comorbid ADHD and 11 had OCD. The analysis showed that youths with comorbid ADHD achieved tic reductions comparable to those without ADHD at post-treatment as well as at 3- and 6-month follow-up assessments. In addition to improvements in tic severity, significant reductions in attentional difficulties, hyperactivity, and impulsivity measured with Conners’ Parent Rating Scale (CPRS) were observed across the entire sample. In contrast, although participants with comorbid OCD also demonstrated clinically meaningful improvements in tic severity, treatment effects were relatively attenuated compared with those observed in individuals without OCD. Overall, these findings indicate that iCBIT remains effective in the presence of common psychiatric comorbidities, particularly ADHD, and may additionally confer benefits on broader behavioural and attentional symptoms [48].

Extending this line of research, Haas et al. [49] conducted the largest RCT to date evaluating a fully therapist-independent, internet-delivered CBIT in adults with chronic tic disorders. In this multicenter, observer-blind study, 161 adults were randomized to receive iCBIT in a group of 67 participants, placebo in 70 participants, or F2F CBIT in 24 participants over 8 weeks. Although the primary endpoint narrowly missed statistical significance immediately after treatment, iCBIT showed a clear trend toward greater tic reduction measured with YGTSS-TTS compared with the placebo condition. In addition, the difference in tic reduction between iCBIT and placebo increased, resulting in a significant difference at the 3- and 6-month follow-ups (2.25 and 2.71 points in the YGTSS-TTS, respectively). Importantly, iCBIT was found to be non-inferior to F2F CBIT, achieving comparable reductions in tic severity despite requiring no direct therapist involvement. Treatment effects continued to strengthen over time, and no treatment-related safety concerns were reported.

All studies examining internet-based delivery of BT for tic disorders are summarized in Table 2.

## 4. Discussion

Digitally supported BT has emerged as a promising extension of established treatments for tic disorders. Although the available evidence remains limited and heterogeneous, existing RCTs suggest that videoconference-based and internet-delivered interventions, such as CBIT, ERP, and HRT, are effective in the treatment of chronic tic disorders and lead to statistically significant reductions in tic severity comparable to F2F BT across pediatric and adult populations. Moreover, these interventions were generally superior to waitlist or psychoeducational control conditions [34,50]. To date, these approaches have demonstrated strong feasibility in the context of specialized tic disorder clinics [37]. Additionally, they are associated with high treatment acceptability and adherence with low dropout rates [41]. From a healthcare systems perspective, digital BT has substantial potential to reduce barriers to access. Internet-delivered interventions can decrease therapist time per patient while enabling the same workforce to treat a larger number of individuals. Economic analyses suggest that remotely delivered BT may be more cost-effective than standard care [45].

To date, only 11 RCTs have evaluated the effectiveness of remotely delivered BT, and the overall sample size remains limited. Although the available results suggest efficacy, not all studies reached their primary endpoints [49]. In addition, some studies lack sufficient statistical power to detect significant between-group effects [32,35]. Non-controlled studies provide additional data; however, they are highly prone to bias, as the absence of a comparator group prevents the isolation of intervention effects from other external factors. Another limitation is that most studies focus on short-term outcomes (8–10 weeks), while long-term data remain scarce. Only a small number of studies report follow-up at 12 or 18 months, although these generally demonstrate sustained reductions in tic severity. Evidence in adult populations is also less extensive than in pediatric samples. Furthermore, potential moderators of treatment response, such as psychiatric comorbidities (e.g., ADHD, OCD, and anxiety), have not been systematically examined, although available data suggest that remote BT remains beneficial in the presence of these comorbid conditions. Another important aspect that is not addressed across studies is the presence of parental support. Parent–child dynamics may play an important role in remote BT, depending on the developmental stage [30]. Methodological challenges inherent to behavioural trials, including limited blinding and frequent allowance of concurrent pharmacotherapy, should also be considered when interpreting results. Another unresolved question concerns the relative effectiveness of different BT approaches when delivered digitally. It remains unclear whether CBIT, ERP, or HRT offers superior outcomes in internet- or teleconference-based formats, as comparative studies directly addressing this question are currently lacking. Taken together, these limitations highlight the importance of cautious interpretation of the reported efficacy.

With regard to future directions, several ongoing studies are expected to further strengthen the evidence base for digitally delivered BT. These include RCTs evaluating internet-delivered interventions as well as feasibility studies of adapted digital treatment programmes within clinical settings [51,52].

## 5. Conclusions

In conclusion, digitally delivered BT represents a promising and increasingly relevant approach for the management of tic disorders, with the potential to substantially improve access to evidence-based care and reduce the treatment gap across pediatric and adult populations. Current evidence supports its feasibility, acceptability, and capacity to reduce tic severity across a range of delivery formats, including videoconference-based therapy, internet-delivered programmes, and app-supported interventions. However, the available evidence remains heterogeneous and should be interpreted with caution, given the variability in study designs, control conditions, intervention formats, and sample sizes. In addition, long-term data remain limited, evidence in adults is still relatively scarce, and the comparative effectiveness of CBIT, ERP, and HRT in digital formats is not yet clear.

For clinical implementation, digital platforms offer a practical opportunity to address long waiting lists, geographical disparities, and the limited availability of specialized services. At the same time, successful implementation requires structured clinical frameworks, including therapist training, supervision, standardized manuals, and clear communication strategies to ensure treatment fidelity and optimize outcomes. Most currently available interventions still rely on therapist or caregiver support, whereas fully automated approaches remain limited. Future research should therefore prioritize larger RCTs, longer follow-up periods, direct comparisons between digital treatment modalities, and systematic evaluation of moderators such as age, comorbidity, symptom profile, and parental involvement. Ongoing studies of internet-delivered and hybrid treatment models are expected to further strengthen the evidence base and support the integration of digital behavioural therapy into routine clinical care.

## Figures and Tables

**Table 1:** Studies examining video-based and videoconference-based delivery of behavioural therapy for tic disorders. Studies are presented in chronological order.

Study (Year)	Study Design	Participants (*n*) /Comparator Group	Age of Participants	Therapy Type (Delivery Format)	Duration of Therapy	Tic and Comorbidities Results
Himle et al., 2012 [32]	RCT	10 VC/10 F2F	8–17 years	CBIT (videoconference)	8 sessions/10 weeks	Mean YGTSS-TSS ↓ 7.8 (32%) in the VC vs. 6.5 (27%) in the F2F group at 4-month follow-up
Ricketts et al., 2016 [33]	RCT	12 VC/8 WL	8–16 years	CBIT (videoconference)	8 sessions/10 weeks	Mean YGTSS-TTS ↓ 7.25 (28.2%) in the VC group vs. 1.75 (8%) in the WL group at post treatment
Singer et al., 2020 [35]	RCT	33 video group/11 F2F	7–13 years	HRT (video-based)	8 sessions/10 weeks	Mean YGTSS-TSS ↓ 9 points (32.4%) in the VC vs. 7.5 points (26.6%) in the F2F at post treatment
Prato et al., 2022 [36]	RCT	20 VC/20 F2F	9–16 years	HRT or ERP (videoconference)	8 sessions/10 weeks	Mean YGTSS ↓ 11.4 points (44.7%) in the VC vs. 12.1 (46.8%) in the F2F Greater CDI reduction (23.6%) in VC; OCD and anxiety improved in both at 2-month follow-up
Capriotti et al., 2023 [37]	Single-arm open trial	29 VC/no control	5–65 years	CBIT (videoconference)	8 sessions/10 weeks	Mean YGTSS-TSS ↓ 6.84 points (25.95%) in pediatric population; mean YGTSS-TSS ↓ 4.5 points (22%) in adults at post treatment
Inoue et al., 2022 [38]	Case series	3 VC/no control	6–13 years	Group-CBIT (videoconference)	8 sessions/10 weeks	Mean YGTSS-TTS ↓ 7.0 points (24.7%) at post treatment
Soerensen et al., 2023 [39]	Non-randomized comparative study	44 VC/72 F2F	6–17 years	ERP (videoconference)	12 weekly sessions	Mean YGTSS-TSS ↓ 11.7 points (46.4%) in the VC group and 10.1 (46.8%) in the F2F-BT group at post treatment and at 1-year follow-up
Jöhnk et al., 2025 [41]	RCT	16 VC/14 app-based training and therapist consultations	9–17 years	HRT or ERP (videoconference)	8 sessions/15 weeks	YGTSS-TTS ↓ 7.43 points (32.2%) in the VC group vs. 9.25 (35.3%) in the app-based BT at post treatment, further reduction at 2-month follow-up

**Table 2:** Studies examining internet-based delivery of behavioural therapy for tic disorders. Studies are presented in chronological order.

Study (Year)	Study Design	Participants (*n*) /Comparator Group	Age of Participants	Therapy Type	Duration of Therapy	Main Tic Results
Andrén et al., 2019 [42]	RCT	12 ERP/11 HRT	8–16 years	BIP TIC ERP vs. BIP TIC HRT	10 weeks	Mean YGTSS-TTS ↓ 6.8 points (28.8%) in the ERP group and 4.1 (17.4%) in the HRT group at 12-month follow-up
Hollis et al., 2021 [50] (ORBIT)	RCT	112 ERP/112 psychoeducation	9–17 years	Internet-delivered ERP vs. psychoeducation	10 weeks	Mean YGTSS-TTS ↓ 4.5 points (16%) in the ERP group vs. 1.6 points (6%) in the psychoeducation group at 3-month follow-up; effects increased at 6 months
Andrén et al., 2022 [43]	RCT	111 ERP/110 psychoeducation	9–17 years	Internet-delivered ERP vs. psychoeducation	10 weeks	Mean YGTSS-TTS ↓ 6.08 (27.3%) points in the ERP vs. 5.29 points (23%) in the psychoeducation at 3-month follow-up; effects stable at 12 months
Rachamim et al., 2022 [47]	Randomized crossover trial	25 iCBIT/16 WL	7–18 years	Internet-delivered CBIT vs. WL	9–10 weeks	Mean YGTSS-TTS ↓ 6.6 points (29%) in the iCBIT vs. 0.94 points (4.3%) in the WL arm at post treatment; effects maintained at 6-month follow-up
Haas et al., 2022 [49]	RCT	67 iCBIT/70 placebo/24 F2F	18–62 years	Fully therapist-independent iCBIT vs. placebo vs. F2F CBIT	8 weeks	Mean YGTSS-TTS ↓ 3.69 points (15.2%) in the iCBIT group vs. 1.44 points (6%) in the placebo group at 3-month follow-up; effects increased at 6 months, iCBIT non-inferior to F2F CBIT
Hollis et al., 2023 [46] (ORBIT follow-up)	RCT	112 ERP/112 psychoeducation	9–17 years	Internet-delivered ERP vs. psychoeducation	10 weeks	Mean YGTSS-TTS reduction maintained in both groups at 12- and 18-month follow-up
Rachamim et al., 2021 [48] (secondary analysis)	Randomized crossover trial	38 CBIT	7–18 years	Internet-delivered CBIT	9–10 weeks	Mean YGTSS-TTS reduction maintained at 3- and 6-month follow-up; comparable effects in patients with and without ADHD
Andrén et al., 2024 [44] (follow up)	RCT	111 ERP/110 psychoeducation	9–17 years	Internet-delivered ERP vs. psychoeducation	10 weeks	No further mean YGTSS-TTS reduction between 3- and 12-month follow-up; effects maintained; no between-group differences

## Data Availability

No new data were created or analyzed in this study. Data sharing is not applicable to this article.

## Abbreviations

The following abbreviations are used in this manuscript:

AAN	American Academy of Neurology
ADHD	Attention-deficit/hyperactivity disorder
ASD	Autism spectrum disorder
BT	Behavioural therapy
BIP	Barninternetprojektet
CBITs	Comprehensive Behavioural Intervention for Tics
CDI	Child Depression Inventory
CY-BOCS	Children’s Yale–Brown Obsessive Compulsive Scale
DBS	Deep brain stimulation
ERP	Exposure and response prevention
ESSTS	European Society for the Study of Tourette Syndrome
F2F	Face to face
HRT	Habit Reversal Training
MASC	Multidimensional Anxiety Scale for Children
OCD	Obsessive–compulsive disorder
PTQ	Parent Tic Questionnaire
RCT	Randomized control trial
TS	Tourette Syndrome
SIB	Self-injurious behaviour
VC	Videoconference
WL	Waiting list
YGTSS	Yale Global Tic Severity Scale
YGTSS-TSS	Yale Global Tic Severity Scale—Total Tic Score
